# Distinct Somatic Alteration Features Identified by Gene Panel Sequencing in Korean Triple-Negative Breast Cancer with High Ki67 Expression

**DOI:** 10.3390/diagnostics11030416

**Published:** 2021-03-01

**Authors:** Woo Young Sun, Jina Lee, Bong Kyun Kim, Jong Ok Kim, Joonhong Park

**Affiliations:** 1 Department of Surgery, College of Medicine, The Catholic University of Korea, Seoul 06591, Korea; sun2729@catholic.ac.kr (W.Y.S.); ljatempe@naver.com (J.L.); bios777@naver.com (B.K.K.); 2 Department of Pathology, College of Medicine, The Catholic University of Korea, Seoul 06591, Korea; jkim@catholic.ac.kr; 3 Department of Laboratory Medicine, Jeonbuk National University Medical School and Hospital, Jeonju 54907, Korea; 4 Clinical Medicine-Biomedical Research Institute, Jeonbuk National University Hospital, Jeonju 54907, Korea

**Keywords:** triple-negative breast cancer, early detection, Ki-67, *PIK3CA*, *TP53*, gene panel sequencing

## Abstract

This study aimed to clarify the genetic difference between Korean triple-negative breast cancer (TNBC) and other breast cancer (BC) subtypes. TNBC was defined as the absence of hormonal receptors and human epidermal growth factor receptor 2 (HER2) amplification. DNA panel of the Ion Torrent Oncomine Comprehensive Assay (OCA) v3 was performed to identify somatic alteration in 48 specimens. In a total of 102 alterations (37 nonsense, 35 missense, 8 frameshift and 22 amplifications), 30 nucleotide alterations (24 nonsense, 1 missense, and 5 frameshift) were newly identified. The eight most commonly altered genes were *PIK3CA*, *TP53*, *ERBB2*, *BRCA2*, *FANCD2*, *AKT1*, *BRCA1*, and *FANCA*. TNBC had significantly lower mutation frequency in *PIK3CA* (TNBC vs. hormone receptor-positive and HER2-negative BC [HRPBC], *p* = 0.009), but higher mutation frequency in *TP53* (TNBC vs. HRPBC, *p* = 0.036; TNBC vs. hormone receptor-positive and HER2- positive BC [HHPBC], *p* = 0.004). TNBC showed frequently higher Ki-67 expression than any positive BC (*p* = 0.004) due to HRPBC (*p* < 0.001). TNBC with high Ki-67/unmutated *PIK3CA*/mutated *TP53* appears at a younger age (52.2 ± 7.6 years), compared to other subtypes (63.7 ± 11.0 years). TNBC with high Ki-67/unmutated *PIK3CA*/mutated *TP53* may be related to relatively early onset BCThese findings demonstrate the genomic heterogeneity between TNBC and other BC subtypes and could present a new approach for molecular targeted therapy in TNBC patients.

## 1. Introduction

Breast cancer (BC) is the most frequently diagnosed cancer (11.6%) and the leading cause of cancer death (6.6%) among women worldwide [1]. In Korea, 22,395 invasive BC cases were recorded in 2017, making it the fifth most frequent cancer type (9.6%) [2]. Hormone receptor (HR)-positive and human epidermal growth factor receptor 2 (HER2)-negative BC (HRPBC) was the most common (65.9%), followed by HR-positive and HER2-positive BC (HHPBC) (11.7%), HR-negative and HER2-positive BC (HER2PBC, 10.2%), and HR-negative and HER2-negative BC (TNBC, 12.2%) [2]. BC is recognized as a complex and diverse group of neoplastic diseases of the breast with distinct molecular characteristics and clinical outcomes [3]. With the elucidation of the intrinsic subtypes of BC, targeted therapies including endocrine therapy or anti-HER2 therapy have been tailored to the specific pathophysiology of HRPBC or HER2PBC [4]. However, patients with TNBC are not eligible for effective selective hormonal modulators or anti-HER2 therapy. The lack of expression of HR and HER2 amplification in TNBC makes it an orphan disease when considering standard therapeutic regimens for BC. An alternative classification divides TNBC into basal-like 1 (BL1), BL2, mesenchymal, and luminal androgen receptor [5]. Even though these subgroups can be further stratified through multi-omics approaches [6], they are largely unknown. Inherited mutations in the *BRCA1* and *BRCA2* genes as tumor suppressor lead to basal-like BC [7], and many TNBC with intact *BRCA*1/2 are classified as BRCAness lesions [8]. Combined loss of the TP53 was identified in 30 to 40% of sporadic TNBC [9]. Additional alterations include *PIK3CA* mutation or PTEN loss and/or enhanced EGFR, WNT and MYC signaling. *TP53*, *PTEN*, and *RB1* are also the most frequent drivers of metastasis in diverse types of human solid cancers including breast cancer [10]. TNBC has been known to be related with a worse prognosis and lower survival rate than other BC subtypes [11]. Even though PARP inhibitors have been approved for chemotherapy of *BRCA1*/*2* mutant/BRCAness TNBC, emergence of clones that resist PARP inhibition through multiple mechanisms is a crucial clinical problem [12]. Considerable racial variations in clinical manifestation and incidence of TNBC have been reported, likely due to the diverse nature of the disease [5,13]. Moreover, the histologic differences of TNBC as well as the heterogeneity in the clinical manifestation may be related to variations in molecular background [14].

The present study aimed to compare the mutation characteristics of BC among its subtypes to clarify the genetic difference between TNBC and other BC subtypes in the Korean population, using comprehensive cancer panel sequencing.

## 2. Materials and Methods

### 2.1. Patients and Specimens

We evaluated 48 patients diagnosed with primary BC and treated at the Department of Surgery, Daejeon St. Mary’s Hospital (Daejeon, Korea) between January 2015 and December 2019. Primary tumor tissues and lymph nodes were surgically resected as standard institutional treatment, and specimens were examined by a board-certified pathologist. The tissues were dissected for histological diagnosis and immunohistochemistry (IHC) staining and then archived as formalin-fixed paraffin-embedded (FFPE) specimens. IHC staining was performed to determine estrogen receptor (ER), progesterone receptor (PR), HER2, and Ki-67 status. The relevant cut-off value for high expression of Ki-67 was defined as 30% described elsewhere [15]. Positive for HER2 is either IHC HER2 3+ (defined as uniform intense membrane staining of > 30% of invasive tumor cells) or fluorescence in situ hybridization (FISH) amplified (ratio of HER2 to CEP17 of >2.2 or average HER2 gene copy number >6 signals/nucleus for those test systems without an internal control probe according to ASCO/CAP guideline recommendations. As a result, 48 BC specimens including 14 with HRPBC, 9 with HER2PBC, 11 HHPBC, and 14 TNBC were selected in this study. Additionally, HRPBC, HER2PBC, and HHPBC were categorized as any positive BC. The FFPE specimens that had more than 50% tumor content to be analyzed in this study were sectioned into 10 micrometers using a new blade and preserved in 1.5 mL Eppendorf tubes. Blade was changed for every tissue block to prevent the contamination of DNA.

### 2.2. DNA Extraction and Purification

Four or five unstained FFPE sections (1 mm thick) were deparaffinized and used for DNA extraction. DNA extraction and purification were performed using the RecoverAll Total Nucleic Acid Isolation Kit for FFPE (ThermoFisher Scientific, Waltham, MA, USA). Extracted DNA was quantified using Qubit 2.0 Fluorometer with Qubit dsDNA HS Assay kit and the TaqMan RNase P Detection Reagents kit (ThermoFisher Scientific, Waltham, MA, USA) and was considered appropriate when the nucleic acid concentration was >10 ng/μL.

### 2.3. Library Preparation for DNA Panel

DNA panel of the Ion Torrent Oncomine Comprehensive Assay (OCA) v3 (ThermoFisher Scientific) was used. The OCA v3 allows concurrent analysis of DNA and RNA to simultaneously detect multiple types of variants across 161 genes relevant to solid tumors, including hotspots, single nucleotide variants (SNVs), insertions and deletions (Indels), copy number variants (CNVs), and gene fusions, in a single workflow. Briefly, 20 ng of the genomic DNA were used in two target amplification reactions, which were then combined. Library preparation for each specimen was performed using the Ion Ampliseq Library Kit 2.0 plus (ThermoFisher Scientific, Waltham, MA, USA) according to the manufacturer’s instructions. The prepared libraries for the DNA panel were partially digested and phosphorylated using the FuPa reagent, ligated to different barcode adapters using the Ion Xpress Barcode Adapters 1–48 Kit (ThermoFisher Scientific, Waltham, MA, USA), then purified. The purified libraries were quantified using the Ion Library TaqMan Quantitation Kit (ThermoFisher Scientific, Waltham, MA, USA).

### 2.4. Sequencing Analysis Using the Ion S5XL

Pooled purified libraries of eight multiplexed tumor DNAs per 540 chip at a concentration of 50 pM were used for chip loading on the Ion Chef with the Ion 540 chef Kit (ThermoFisher Scientific, Waltham, MA, USA) and subsequently sequenced on S5XL using Ion S540 chip (ThermoFisher Scientific, Waltham, MA, USA) as per the manufacturer’s instructions.

### 2.5. Sanger Sequencing

Because somatic tumor alterations were identified without matched normal specimens, mutation origin with allele frequency of near 50% (heterozygous) or 100% (homozygous) as possible germline origin was determined by Sanger sequencing using matched germline DNA from peripheral blood. Additionally, Sanger sequencing was performed to confirm some of the detected alterations with mutant allele burden >15%. Capillary electrophoresis was performed on the 3730XL Genetic Analyzer (Applied Biosystems, Carlsbad, CA, USA). Sequence data were analyzed using the Sequencher DNA Sequence Analysis Software Demo Version 4.9 (Gene Codes Corporation, Ann Arbor, MI, USA).

### 2.6. Bioinformatic Analysis

Analysis of sequencing raw data was performed by Torrent Suite software ver 5.10 (ThermoFisher Scientific, Waltham, MA, USA) using default analysis parameters. Data analyses for variant calling (SNVs/multi-nucleotide variants [MNVs], indels, and CNVs) were performed in Ion Reporter software ver 5.10. Available online: https://ionreporter.thermofisher.com/ir/ (accessed on 11 November 2020) with Torrent Variant Caller and Coverage Analysis plug-ins using default settings. A minimum sequencing depth of 500× was considered as adequate sequencing depth, and an allelic frequency of 5% was used as a cut-off for variants. Human genome build 19 was used as the reference for alignment. Briefly, annotation of the results, filtering of spurious and repeat errors, and interface for visualization of sequencing reads via Integrative Genome Viewer were performed using software built in-house, as previously described [16]. Mutations predicted to cause strong and moderate alteration on gene functions, such as stop gained/lost, initiator codon, missense, frameshift, and splice site mutation were manually reviewed by laboratory geneticists based on ACMG-AMP standards and guidelines [17]. Particularly, multiple missense functional predictors including SIFT, polyphen2, MutationTaster, and MutationAssessor were used to determine their deleterious effect. In addition, genes that play a role as either oncogene or tumor suppressor based on their typical behavior in cancer were assessed using ClinVar. Available online: https://www.ncbi.nlm.nih.gov/clinvar/ (accessed on 11 November 2020), Online Mendelian Inheritance in Man. Available online: https://www.omim.org/ (accessed on 11 November 2020), and Catalogue of Somatic Mutations in Cancer. Available online: https://cancer.sanger.ac.uk/cosmic (accessed on 11 November 2020). When the number of gene roles in cancer is more than half, it is defined as either oncogene or tumor suppressor dominance. On the other hand, data analyses for identifying copy number variations were performed in Ion Reporter software v5.10 with a baseline from the average sequencing depths achieved in a set of normal DNA from 16 peripheral blood specimens and manufacturer recommended settings. The copy numbers for genes in a given specimens were calculated by comparing the average sequencing depth achieved by the amplicons covering the gene in the specimen to the historical sequencing depths (baseline) by using the algorithm previously described [18].

### 2.7. Statistical Analysis

Descriptive statistics were used to show the mean ± SD of age and BMI of studied population. To compare somatic alteration profiles between BC subtypes, genomic characteristics were compared across cohorts using one-way analysis of variance followed by Scheffe’s post hoc test for continuous variables. The difference of somatic alterations was estimated using Fisher’s exact test. A two-tailed *p* < 0.05 was considered to indicate a statistically significant difference. Statistical analysis was performed using MedCalc Statistical Software Version 19.5.3 (MedCalc Software Ltd., Ostend, Belgium).

## 3. Results

### 3.1. Clinicopathological Features of Studied Patients

All 48 patients were female, and four had a family history of BC. The mean age ± standard deviation (SD) at diagnosis was 62.4 ± 11.2 years (range, 43–81 years). The mean body mass index was 24.7 ± 3.6 kg/m^2^. The majority of patients (73%) had early-stage breast cancer (stage Ia, 42% (20/48); stage IIa, 31% (15/48)). All patients were followed up for up to 3 years. No clinical relapse or cancer-related death occurred during the follow-up period. The clinicopathological features of the patients are shown in Table 1.

### 3.2. Quality Control Metrics of Raw Sequencing Data

In quality control (QC) metrics for raw sequencing data generated from six independent experiments, the mean of total usable reads was 89,630,464 (63.8%) and the mean of read length was 105 bp (SD, 6; range, 96–111). The mean of mapped read count, on-target read rate, the mean depth of on-target regions, and uniformity were 11,299,984, 95.4%, 2976×, and 93.3%, respectively. Overall, all experiments satisfied the manufacturer’s specifications (>95% of amplicons should have a read depth >500×).

### 3.3. Somatic Alteration Profiles

**Figure 1:**
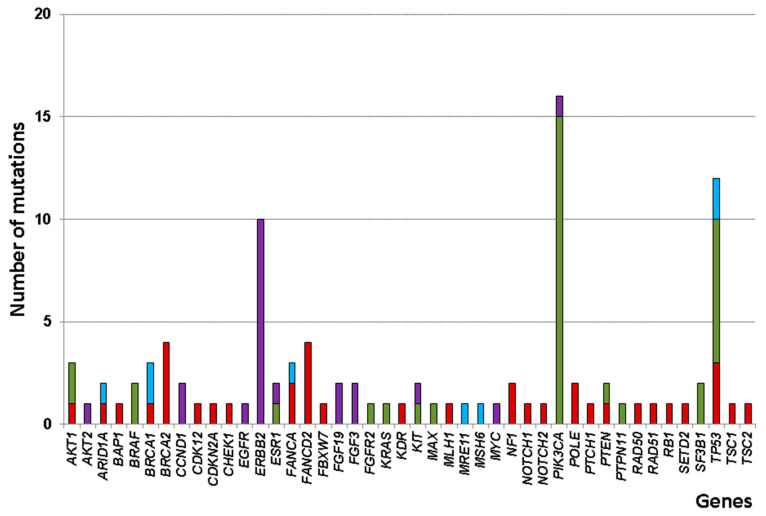
Frequencies of somatic alteration profiles in various genes identified using the Oncomine comprehensive v3 DNA assay in 41 patients with breast cancer. Genes are depicted on the x-axis, and the number of alterations is indicated on the y-axis. Green, missense mutation; red, nonsense mutation; blue, frameshift mutation; violet, amplification.

A total of 11,088 unfiltered variants were identified from the raw sequencing data using the OCA v3 DNA assay. We analyzed the filtered cancer driver genes to determine potential genes of interest. After variant filtering with Oncomine Comprehensive v3/w4.0/DNA/Single Sample workflow, 80 somatic SNVs or indels and 22 CNVs passed data analysis algorithms. Of the 80 somatic mutations, 37 nonsense, 35 missense, and 8 frameshift mutations were identified. Thirty nucleotide alterations (24 nonsense, 1 missense, and 5 frameshift) were newly identified. The p.His28Tyr of *MAX* gene is predicted to be deleterious based on missense functional predictors (SIFT, deleterious (Score 0); Polyphen2, probably damaging (1.00); MutationTaster, disease causing (83); and MutationAssessor, high (3.57)). The eight most commonly altered genes were *PIK3CA*, *TP53*, *ERBB2*, *BRCA2*, *FANCD2*, *AKT1*, *BRCA1*, and *FANCA* (Figure 1). We examined genes altered in multiple specimens and found that three genes were altered in at least 20% of these specimens. *PIK3CA* was the most frequently altered gene, with variants found in 15 HRPBC or HHPBC specimens. The second most frequently altered gene was *TP53*, which was shared by 12 specimens. Interestingly, several oncogenic *TP53* mutations in 6 out of 11 TNBCs, including p.Lys132Gln, p.Pro177_Cys182del, p.Gln192*, p.Glu204*, p.His214Arg, p.Tyr236Cys, p.Cys238*, p.Arg248Trp, p.Arg273His, and p.Pro278Leu were detected in 6 TNBC. We also found several recurrent mutations in other genes, including the previously reported mutations in p.Glu17Lys of *AKT1* (2/3, 66%), p.Lys812Argfs*3 of *BRCA1* (2/3, 66%; germline), and p.Lys700Glu of *SF3B1* (2/2, 100%) (Supplementary Table S1). Meanwhile, of the 13 specimens, all 22 CNVs in 10 different genes were amplified. Interestingly, *ERBB2*, the third most frequently altered gene, was only amplified but not mutated in 10 HER2PBC or HHPBC specimens (Supplementary Table S2). We also found several CNVs in other genes: Two in *CCND1*, *FGF3*, and *FGF19*. One in *AKT2*, *EGFR*, *ESR1*, *KIT*, *MYC*, and *PIK3CA*.

**Figure 2:**
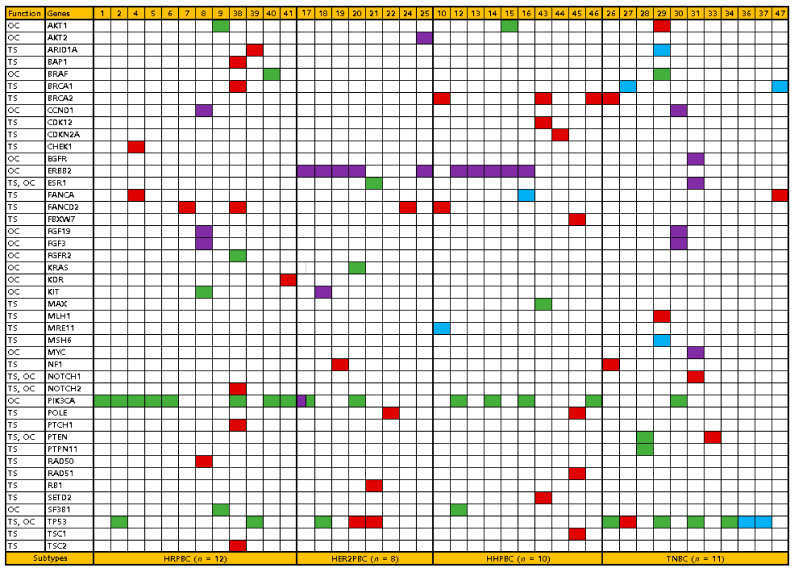
Distribution of somatic alteration profiles based on breast cancer subtypes in 41 patients with breast cancer. Each patient is depicted on the x-axis, and the genes are indicated on the y-axis. Green, missense mutation; red, nonsense mutation; blue, frameshift mutation; violet, amplification. HRPBC, hormone receptor-positive breast cancer; HER2PBC, hormone receptor-negative HER2-positive breast cancer; HHPBC, hormone receptor-positive and HER2-positive breast cancer; TNBC, triple-negative breast cancer. OC, oncogene; TS, tumor suppressor.

Collectively, we found an average of 2.5 alterations per specimen (range, 1–8 alterations) in the 44 unique cancer driver genes (Figure 2). Twenty-five of the 41 specimens had an alteration in *TP53* and/or *PIK3CA*. The most commonly recurring mutation was the p.His1047Arg of *PIK3CA*, which was found in the majority of the *PIK3CA* mutant specimens (8/15, 53%), followed by p.Asn345Lys (2/15, 13%) and p.Glu542Lys of *PIK3CA* (2/15, 13%).

### 3.4. Comparison of Somatic Alteration Profiles between Breast Cancer Subtypes

The presence of alteration and alteration type (nucleotide change vs. CNV) were not statistically significant between TNBC and any positive BC. Of three frequent alterations, PIK3CA and TP53 alterations were statistically significant between TNBC and any positive BC, respectively. Compared to the somatic alteration profiles of other BC subtypes, TNBC had significantly lower mutation frequency in *PIK3CA* (TNBC vs. HRPBC, *p* = 0.009), but higher mutation frequency in *TP53* (TNBC vs. HRPBC, *p* = 0.036; TNBC vs. HHPBC, *p* = 0.004). Interestingly, significant differences in gene role dominance were observed between TNBC and HRPBC (*p* = 0.024) as well as between TNBC and any positive BC (*p* = 0.010) (Table 2).

### 3.5. Ki-67 Expression and PIK3CA/ TP53 Mutation Status

TNBC showed frequently higher Ki-67 expression than any positive BC (*p* = 0.004) due to HRPBC (*p* < 0.001). Higher Ki-67 expression was not statistically different compared to HHPBC (*p* = 0.081) and HER2PBC (*p* = 1.000). PIK3CA and/or TP53 were the most commonly mutated gene in this study, with 25 of the 41 specimens (61%) containing a mutation. To identify the roles of *PIK3CA* and/or *TP53* and its association with Ki-67 expression, we subdefined TNBC with high Ki-67/unmutated *PIK3CA*/mutated *TP53* subdivided from TNBC subtype. Six (specimens 26, 27, 31, 34, 36, and 37) of the 14 TNBC specimens showed high Ki-67/unmutated *PIK3CA*/mutated *TP53* TNBC subtype. Interestingly, TNBC with high Ki-67/unmutated *PIK3CA*/mutated *TP53* appeared at a younger age (52.2 ± 7.6 years) than any positive BC (63.7 ± 11.0 years). However, there was no significant difference in the age of onset between TNBC and each BC subtype or any positive BC. Furthermore, there was no association among *PIK3CA* and/or *TP53* mutation status, Ki-67 expression, and cancer staging.

## 4. Discussion

Earlier onset, aggressive tumor phenotype, and more advanced stage at diagnosis are distinct features of TNBC in women with African ethnicity compared to Caucasians, denoting one of the most characteristic findings of racial disparity in cancer oncology [19]. However, the higher frequency of TNBC in African Americans is not related to a different genomic profile of commonly established tumor regulatory pathway genes [20]. In multiracial early onset TNBC study, African Americans had the highest number of deleterious mutations compared to European Americans, Hispanic, and Asian populations. However, the trend was reversed such that African Americans carried fewer mutations when focusing on mutations in the known breast cancer genes [21]. Thus, further classification of TNBC, considering ethnicity/genetic background, is required to in order to detect early neoplastic changes; thus, facilitating the detection of TNBC at an early stage is one of the most important challenges in the treatment of BC. In this study, we demonstrate that the predictive potential of Ki-67, *PIK3CA*, and *TP53* status in relatively early onset Korean TNBC, compared to other BC subtypes. Combining somatic alteration profiles and Ki-67 state, TNBC with high Ki-67/unmutated *PIK3CA*/mutated *TP53* appeared at a younger age than any positive BC. Of eight Korean BC under the age of 50 years, four were high Ki-67/unmutated *PIK3CA*/mutated *TP53* TNBC with stage 2A/B (*n* = 3) and 1A (*n* = 1). Ki-67 expression over 30% was significantly associated with worse prognosis, especially for stage I patients [15].

Similar to previous studies [14,22,23], our study demonstrated that *TP53* is the most commonly mutated gene, but *PIK3CA* mutation is rare in TNBC. In BC, oncogenic *TP53* mutations in the DNA-binding domain from codon sequences 102 to 292 are related to poor prognosis compared to wild-type *TP53*, which is associated with better clinical outcome in BC [24]. However, the impact of oncogenic *TP53* mutations on the overall survival and disease-free survival for TNBC was not available, because all patients were alive without disease recurrence during our 3-year follow-up period. A previous study reported that a subset of TNBC harbors somatic mutations in the genome repair system [25]. In our study, several tumor suppressor genes such as not only *BRCA1/2* and *TP53* but also *MLH1*, *MSH6*, *NF1*, and *PTPN11* were predominantly altered in TNBC. However, there were no predominant mutated genes or hotspot mutations. A higher mutational burden of tumor was also more commonly observed in HR-negative BC than in HRPBC [26]. More deleterious mutations in multiple genes, including *BRCA1*, *BRCA2* and other predisposition genes, are associated with TNBC [27]. In this study, deleterious alterations such as nonsense or frameshift were slightly high in TNBC (57%, 8/14) than in any positive BC (50%, 17/34), which were not statistically significant (*p* = 0.756). Similarly, multiple alterations with ≧ 4 in one specimen were observed in TNBC (21%, 3/14) than in any positive BC (15%, 5/34), which was not statistically significant (*p* = 0.676).

*PIK3CA* mutations are usually enriched in 29 to 45% of HR-positive tumors, with a lower frequency in TNBC [28]. Similar to other breast cancer studies, *PIK3CA* was the most commonly mutated oncogene (31%, 15/48) in our study. The mutational incidence of *PIK3CA* in TNBC was much lower (7%, 1/14) in this study than that in previously published research [14,22,23]. However, dysregulation of signaling through the PI3K and AKT signaling pathways is one of the most common oncogenic aberrations in TNBC. Selecting patients for AKT inhibition according to *PI3KCA*/*AKT1*/*PTEN* alterations appears to optimize the treatment outcomes [29].

Most TNBC patients do not carry germline mutations of *BRCA1* or *BRCA2*, which are critical for maintaining genome integrity. However, pathological high-grade BCs and TNBC often show somatic mutations or abnormal BRCA1 or BRCA2 expression [30]. In our study, two TNBC specimens with p.Lys812Argfs*3 germline *BRCA1* mutation were identified. BCs occurring in most germline *BRCA1* mutation carriers are TNBCs. In contrast, there is no specific breast cancer subtype in *BRCA2* carriers [31]. Defects in the genome repair machinery associated with *BRCA1* and *BRCA2* mutations could optimize the treatment outcomes of platinum-based chemotherapy or PARP inhibitors in these patients [32].

Alternative pathways in cell proliferation, differentiation, apoptosis, and invasion, such as paracrine/autocrine loops of growth factors, have been suggested as novel candidate therapeutic pathways and targets in TNBC. SHP-1/p-STAT3/VEGF-A axis is a potential therapeutic target for metastatic TNBC, and the more potent SC-78 may be a promising lead for suppressing metastasis of TNBC [33]. Sequential combination of docetaxel with a SHP-1 agonist enhanced suppression of p-STAT3 signaling and apoptosis in triple negative breast cancer cells [34]. Targeting SHP-1/p-STAT3 and the potential combination of SHP-1 agonist with chemotherapeutic docetaxel is a feasible therapeutic strategy for TNBC [35].

Our study had some limitations. First the number of studied patients was small, and the patients were from a single center, although each BC subtype was well classified. Second, comprehensive cancer panel sequencing was performed on only tumor DNA extracted from FFPE specimens. The quality of tumor DNA from FFPE is lower than that from fresh specimens, potentially causing variant call discrepancies [36]. A low tumor allele frequency below our cut-off of 5% carrying “actual” mutations could also be missed. Third, the result of targeted gene panel sequencing is affected by the genomic size targeted by the panel and by its gene composition. To resolve this discrepancy, core genes established to have diagnostic, therapeutic, or prognostic relevance in BC should be included when designing gene panels. Fourth, the short follow-up period of up to 3 years was inadequate to observe clinically meaningful associations between genomic alterations and clinicopathological features.

## 5. Conclusions

In conclusion, our study demonstrates that TNBC with high Ki-67/unmutated *PIK3CA*/mutated *TP53* may be related to relatively early-onset BC. The somatic alteration profiles from Korean TNBC patients were found to contribute to the mutation characteristics of Asian BC patients, providing insights into the genome landscape of BC and further evidence on the role of Ki-67, *PIK3CA*, and *TP53* in breast carcinogenesis. These findings demonstrate the genomic heterogeneity between TNBC and other BC subtypes and could present a new approach for molecular targeted therapy in TNBC patients.

## Figures and Tables

**Table 1:** Comparison results of clinicopathological features in 48 Korean patients with breast cancer.

Features	Total (*n* = 48)	HRPBC (*n* = 14)	HER2PBC (*n* = 9)	HHPBC (*n* = 11)	TNBC (*n* = 14)	*p* Value *
Age (Mean ± SD), year	62.4 ± 11.2	64.4 ± 11.0	63.6 ± 10.7	63.1 ± 12.1	59.1 ± 11.4	0.638
<50	7	1	1	1	4	0.365
≥50	41	13	8	10	10	
Familial history	4	1	0	0	3	0.173
Postmenopause	41	13	8	10	10	0.365
BMI (Mean ± SD), kg/m^2^	24.7 ± 3.6	24.9 ± 3.0	23.7 ± 4.5	24.8 ± 4.0	25.0 ± 3.6	0.846
Primary tumor size						0.402
1	24	9	3	4	8	
2	22	4	6	6	6	
3	1	1	0	0	0	
4	1	0	0	1	0	
Lymph node metastasis						0.601
0	33	9	6	7	11	
1	8	1	1	3	3	
2	4	2	1	1	0	
3	3	2	1	0	0	
Pathologic stage						0.214
I	20	7	2	3	8	
II	20	3	5	6	6	
III	8	4	2	2	0	
Ki67, %						0.004
<30	23	13	2	6	2	
≥30	25	1	7	5	12	
Type of breast surgery						0.590
Breast conservation	29	10	4	6	9	
Total mastectomy	19	4	5	5	5	
Type of axillary surgery						0.757
Sentinel node biopsy	34	9	7	7	11	
Axillary dissection	14	5	2	4	3	
Adjuvant chemotherapy	40	8	9	9	14	0.009
Adjuvant radiotherapy	36	13	5	7	11	0.168
Hormone therapy	25	14	0	11	0	<0.001
HER2 target therapy	17	0	8	9	0	<0.001

HRPBC, hormone receptor positive breast cancer; HER2PBC, HER2 positive breast cancer; HHPBC, hormone receptor positive and HER2 positive breast cancer; TNBC, triple negative breast cancer; * *p*-value indicates statistical significance between TNBC and any positive BC by Fisher’s exact test.

**Table 2:** Comparison results of somatic alteration profiles in 48 Korean patients with breast cancer.

Features	Total (*n* = 48)	HRPBC (*n* = 14)	HER2PBC (*n* = 9)	HHPBC (*n* = 11)	TNBC (*n* = 14)	*p* Value *
Alteration number						0.942
0	7	2	1	1	3	
1	12	4	2	2	4	
≥2	29	8	6	8	7	
Alteration type						0.983
Missense	35	15	5	7	8	
Nonsense	37	12	6	11	8	
Frameshift	8	0	0	2	6	
Amplification	22	3	8	5	6	
Frequent alteration						
*ERBB2*	10	0	5	5	0	0.085
*PIK3CA*	15	8	2	4	1	0.037
*TP53*	12	2	3	0	7	0.024
Gene role dominance						0.010
Oncogene	17	7	4	5	1	
Tumor suppressor	20	4	3	4	9	
Codominant	4	1	1	1	1	

HRPBC, hormone receptor positive breast cancer; HER2PBC, HER2 positive breast cancer; HHPBC, hormone receptor positive and HER2 positive breast cancer; TNBC, triple negative breast cancer; * *p*-value indicates statistical significance between TNBC and any positive BC by Fisher’s exact test.

## Data Availability

The data presented in this study are available on request from the corresponding author.

## Supplementary Materials

The following are available online at https://www.mdpi.com/2075-4418/11/3/416/s1, Table S1: Details of mutation profiles in 39 Korean patients with breast cancer. Table S2: Details of copy number variations in 13 Korean patients with breast cancer.
