# Supplementary material for: Distinct Somatic Alteration Features Identified by Gene Panel Sequencing in Korean Triple-Negative Breast Cancer with High Ki67 Expression
Source: Diagnostics (Basel). 2021 Mar 1;11(3):416. doi: 10.3390/diagnostics11030416 (PMC8000916; doi:10.3390/diagnostics11030416)
Supplement: Supplementary file 1 [file diagnostics-11-00416-s001.pdf]

Supplementary Table S1. Details of mutation profiles in 39 Korean patients with breast cancer

SN	Subtype	Genes	Transcript	Base change	Codon change	Type	AF (%)	COSMIC ID /RS number*
1	HRPBC	*PIK3CA*	NM_006218.4	c.3140A>G	p.His1047Arg	Missense	11.7	COSM775
2	HRPBC	*PIK3CA*	NM_006218.4	c.3140A>G	p.His1047Arg	Missense	30.7	COSM775
2	HRPBC	*TP53*	NM_000546.5	c.641A>G	p.His214Arg	Missense	24.8	COSM43687
4	HRPBC	*CHEK1*	NM_001274.5	c.337C>T	p.Gln113*	Nonsense	12.4	
4	HRPBC	*FANCA*	NM_000135.4	c.1867C>T	p.Gln623*	Nonsense	11.6	
4	HRPBC	*PIK3CA*	NM_006218.4	c.3140A>G	p.His1047Arg	Missense	32.1	COSM775
5	HRPBC	*PIK3CA*	NM_006218.4	c.331A>G	p.Lys111Glu	Missense	18.7	COSM13570
6	HRPBC	*PIK3CA*	NM_006218.4	c.3140A>G	p.His1047Arg	Missense	21.9	COSM775
7	HRPBC	*FANCD2*	NM_033084.5	c.2614C>T	p.Gln872*	Nonsense	11.7	
8	HRPBC	*KIT*	NM_000222.2	c.2387G>A	p.Arg796Lys	Missense	12.7	COSM1600411
8	HRPBC	*RAD50*	NM_005732.4	c.2626C>T	p.Gln876*	Nonsense	11.7	
9	HRPBC	*AKT1*	NM_001014431.2	c.49G>A	p.Glu17Lys	Missense	43.6	COSM33765
9	HRPBC	*SF3B1*	NM_012433.3	c.2098A>G	p.Lys700Glu	Missense	24.9	COSM84677
38	HRPBC	*BAP1*	NM_004656.4	c.587G>A	p.Trp196*	Nonsense	12.1	rs1553645725
38	HRPBC	*BRCA1*	NM_007294.3	c.5542C>T	p.Gln1848*	Nonsense	12.0	rs886040303
38	HRPBC	*FANCD2*	NM_033084.5	c.1684C>T	p.Gln562*	Nonsense	13.0	
38	HRPBC	*FGFR2*	NM_000141.4	c.1145G>A	p.Cys382Tyr	Missense	12.0	COSM915493
38	HRPBC	*NOTCH2*	NM_024408.4	c.5614C>T	p.Gln1872*	Nonsense	12.8	
38	HRPBC	*PIK3CA*	NM_006218.4	c.3133G>A	p.Asp1045Asn	Missense	12.9	COSM25086
38	HRPBC	*PTCH1*	NM_000264.4	c.478C>T	p.Gln160*	Nonsense	11.7	
38	HRPBC	*TSC2*	NM_000548.5	c.1327C>T	p.Gln443*	Nonsense	11.6	
39	HRPBC	*ARID1A*	NM_006015.6	c.5164C>T	p.Arg1722*	Nonsense	11.9	COSM51418
39	HRPBC	*TP53*	NM_000546.5	c.818G>A	p.Arg273His	Missense	20.2	COSM10660
40	HRPBC	*BRAF*	NM_004333.6	c.1793C>T	p.Ala598Val	Missense	11.6	COSM21549
40	HRPBC	*PIK3CA*	NM_006218.4	c.3140A>G	p.His1047Arg	Missense	14.2	COSM775
41	HRPBC	*HNF1A*	NM_000545.6	c.338G>A	p.Trp113*	Nonsense	11.8	
41	HRPBC	*PIK3CA*	NM_006218.4	c.3140A>G	p.His1047Arg	Missense	30.5	COSM775
17	HER2PBC	*PIK3CA*	NM_006218.4	c.3140A>G	p.His1047Arg	Missense	81.5	COSM775
18	HER2PBC	*TP53*	NM_000546.5	c.707A>G	p.Tyr236Cys	Missense	52.8	COSM10731
19	HER2PBC	*NF1*	NM_001042492.2	c.2087G>A	p.Trp696*	Nonsense	11.5	
20	HER2PBC	*KRAS*	NM_033360.4	c.35G>A	p.Gly12Asp	Missense	57.2	COSM521
20	HER2PBC	*PIK3CA*	NM_006218.4	c.1624G>A	p.Glu542Lys	Missense	36.9	COSM760
20	HER2PBC	*TP53*	NM_000546.5	c.610G>T	p.Glu204*	Nonsense	33.1	
21	HER2PBC	*ESR1*	NM_001122740.1	c.1174G>A	p.Val392Ile	Missense	12.5	COSM1545070
21	HER2PBC	*RB1*	NM_000321.2	c.157G>T	p.Glu53*	Nonsense	30.0	
21	HER2PBC	*TP53*	NM_000546.5	c.714delT	p.Cys238*	Nonsense	48.8	
22	HER2PBC	*POLE*	NM_006231.3	c.6550C>T	p.Gln2184*	Nonsense	12.0	
24	HER2PBC	*FANCD2*	NM_033084.5	c.1091G>A	p.Trp364*	Nonsense	6.5	
10	HHPBC	*BRCA2*	NM_000059.3	c.2979G>A	p.Trp993*	Nonsense	11.8	rs80358544
10	HHPBC	*FANCD*	NM_033084.5	c.4381C>T	p.Gln1461*	Nonsense	11.7	
10	HHPBC	*MRE11*	NM_005591.3	c.186delT	p.His63Metfs*17	Frameshift	97.3	
12	HHPBC	*PIK3CA*	NM_006218.4	c.3140A>G	p.His1047Arg	Missense	18.7	COSM775
12	HHPBC	*SF3B1*	NM_012433.3	c.2098A>G	p.Lys700Glu	Missense	37.6	COSM84677
14	HHPBC	*PIK3CA*	NM_006218.4	c.1035T>A	p.Asn345Lys	Missense	45.6	COSM754
15	HHPBC	*AKT1*	NM_001014431.2	c.49G>A	p.Glu17Lys	Missense	47.0	COSM33765
16	HHPBC	*FANCA*	NM_000135.4	c.3719_3723delAAAAC	p.Glu1240Aspfs*36	Frameshift	76.4	
16	HHPBC	*PIK3CA*	NM_006218.4	c.1633G>A	p.Glu545Lys	Missense	53.5	COSM763
43	HHPBC	*BRCA2*	NM_000059.3	c.9739C>T	p.Gln3247*	Nonsense	6.0	rs886040849

43	HHPBC	*CDK12*	NM_016507.4	c.1834C>T	p.Gln612*	Nonsense	6.2	
43	HHPBC	*MAX*	NM_002382.5	c.82C>T	p.His28Tyr	Missense	11.7	
43	HHPBC	*SETD2*	NM_014159.6	c.4933C>T	p.Gln1645*	Nonsense	12.3	
44	HHPBC	*CDKN2A*	NM_001195132.1	c.238C>T	p.Arg80*	Nonsense	11.3	COSM12475
45	HHPBC	*FBXW7*	NM_033632.3	c.661C>T	p.Gln221*	Nonsense	12.9	
45	HHPBC	*TSC1*	NM_000368.4	c.2593C>T	p.Gln865*	Nonsense	8.1	
45	HHPBC	*POLE*	NM_006231.3	c.2324G>A	p.Trp775*	Nonsense	7.0	
45	HHPBC	*RAD51*	NM_133487.4	c.706C>T	p.Arg236*	Nonsense	12.5	
46	HHPBC	*BRCA2*	NM_000059.3	c.9076C>T	p.Gln3026*	Nonsense	52.2	rs80359159
46	HHPBC	*PIK3CA*	NM_006218.4	c.1035T>A	p.Asn345Lys	Missense	27.8	COSM754
26	TNBC	*BRCA2*	NM_000059.3	c.8969G>A	p.Trp2990*	Nonsense	11.6	rs80359148
26	TNBC	*NF1*	NM_001042492.2	c.3916C>T	p.Arg1306*	Nonsense	12.3	rs376576925
26	TNBC	*TP53*	NM_000546.5	c.742C>T	p.Arg248Trp	Missense	24.7	COSM10656
27	TNBC	*BRCA1*	NM_007294.3	c.2433delC	p.Lys812Argfs*3	Frameshift	63.0	rs80357524
27	TNBC	*TP53*	NM_000546.5	c.574C>T	p.Gln192*	Nonsense	78.0	COSM10733
28	TNBC	*PTEN*	NM_000314.7	c.388C>G	p.Arg130Gly	Missense	27.3	COSM5219
28	TNBC	*PTPN11*	NM_002834.4	c.205G>A	p.Glu69Lys	Missense	17.0	COSM13013
29	TNBC	*AKT1*	NM_001014431.2	c.292G>T	p.Glu98*	Nonsense	11.7	COSM7449961
29	TNBC	*ARID1A*	NM_006015.6	c.1057delG	p.Ala353Profs*10	Frameshift	83.2	
29	TNBC	*BRAF*	NM_004333.6	c.1798G>A	p.Val600Met	Missense	7.5	COSM1130
29	TNBC	*MLH1*	NM_000249.3	c.589C>T	p.Gln197*	Nonsense	11.9	rs1553644123
29	TNBC	*MSH6*	NM_000179.2	c.4071_4072insGATT	p.Lys1358Aspfs*2	Frameshift	49.8	
29	TNBC	*TP53*	NM_000546.5	c.856G>A	p.Glu286Lys	Missense	19.4	COSM10726
30	TNBC	*PIK3CA*	NM_006218.4	c.1624G>A	p.Glu542Lys	Missense	12.7	COSM760
31	TNBC	*NOTCH1*	NM_017617.5	c.4699G>T	p.Glu1567*	Nonsense	43.0	
31	TNBC	*TP53*	NM_000546.5	c.394A>C	p.Lys132Gln	Missense	63.4	COSM11224
33	TNBC	*PTEN*	NM_000314.7	c.955_958delACTT	p.Thr319*	Nonsense	42.4	COSM4898
34	TNBC	*TP53*	NM_000546.5	c.833C>T	p.Pro278Leu	Missense	30.7	COSM10863
36	TNBC	*TP53*	NM_000546.5	c.529_546del	p.Pro177_Cys182del	Frameshift	43.5	COSM43570
37	TNBC	*TP53*	NM_000546.5	c.264_270delCCCCTCC	p.Pro89Glyfs*32	Frameshift	34.4	
47	TNBC	*BRCA1*	NM_007294.3	c.2433delC	p.Lys812Argfs*3	Frameshift	71.0	rs80357524
47	TNBC	*FANCA*	NM_000135.4	c.893G>A	p.Trp298*	Nonsense	11.5	

SN, specimen number; COSMIC ID, Catalogue of Somatic Mutations in Cancer ID; RS number, Reference SNP cluster ID

*Alterations without COSMIC ID or RS number are newly identified in this study.

Supplementary Table S2. Details of copy number variations in 13 Korean patients with breast cancer

SN	Subtype	Genes	Type	Length (Kb)	Copy Number	CytoBand
8	HRPBC	*CCND1*	Amplification	10.1	13.84	11q13.3(69455972-69466035)x13.84
8	HRPBC	*FGF3*	Amplification	8.8	16.86	11q13.3(69624976-69633775)x16.86
8	HRPBC	*FGF19*	Amplification	4.8	9.08	11q13.3(69513954-69518740)x9.08
17	HER2PBC	*ERBB2*	Amplification	14.8	13.83	17q12(37868168-37882971)x13.83
17	HER2PBC	*PIK3CA*	Amplification	35.6	16.97	3q26.32(178916549-178952114)x16.97
18	HER2PBC	*KIT*	Amplification	13.7	15.05	4q12(55589728-55603448)x15.05
18	HER2PBC	*ERBB2*	Amplification	14.8	23.62	17q12(37868168-37882971)x23.62
19	HER2PBC	*ERBB2*	Amplification	14.8	12.42	17q12(37868168-37882971)x12.42
20	HER2PBC	*ERBB2*	Amplification	14.8	16.09	17q12(37868168-37882971)x16.09
25	HER2PBC	*AKT2*	Amplification	23.2	9.54	19q13.2(40739755-40762984)x9.54
25	HER2PBC	*ERBB2*	Amplification	14.8	9.93	17q12(37868168-37882971)x9.93
12	HHPBC	*ERBB2*	Amplification	14.8	12.67	17q12(37868168-37882971)x12.67
13	HHPBC	*ERBB2*	Amplification	14.8	10.04	17q12(37868168-37882971)x10.04
14	HHPBC	*ERBB2*	Amplification	14.8	13.4	17q12(37868168-37882971)x13.4
15	HHPBC	*ERBB2*	Amplification	14.8	11.82	17q12(37868168-37882971)x11.82
16	HHPBC	*ERBB2*	Amplification	14.8	12.08	17q12(37868168-37882971)x12.08
30	TNBC	*CCND1*	Amplification	10.1	7.66	11q13.3(69455972-69466035)x7.66
30	TNBC	*FGF3*	Amplification	8.8	8.75	11q13.3(69624976-69633775)x8.75
30	TNBC	*FGF19*	Amplification	4.8	7.82	11q13.3(69513954-69518740)x7.82
31	TNBC	*EGFR*	Amplification	57.1	17.63	7p11.2(55211010-55268091)x17.63
31	TNBC	*ESR1*	Amplification	291.0	15.31	6q25.1(152129033-152420035)x15.31
31	TNBC	*MYC*	Amplification	4.5	15.8	8q24.21(128748724-128753198)x15.8

SN, specimen number
